# Prognostic value of immune checkpoint molecules in breast cancer

**DOI:** 10.1042/BSR20201054

**Published:** 2020-07-07

**Authors:** Jun Fang, Feng Chen, Dong Liu, Feiying Gu, Zhigang Chen, Yuezhen Wang

**Affiliations:** 1 Institute of Cancer and Basic Medicine (ICBM), Chinese Academy of Sciences, Zhejiang, Hangzhou 310022, People’s Republic of China; 2 Department of Radiation Oncology, Zhejiang Cancer Hospital, Cancer Hospital of The University of Chinese Academy of Sciences, Zhejiang, Hangzhou 310022, People’s Republic of China; 3 Key Laboratory of Radiation Oncology of Zhejiang Province, Zhejiang, Hangzhou 310022, People’s Republic of China; 4 Department of Breast Tumor Surgery, Institute of Cancer Research and Basic Medical Sciences of Chinese Academy of Sciences, Cancer Hospital of University of Chinese Academy of Sciences, Zhejiang Cancer Hospital, Hangzhou, People’s Republic of China; 5 Department of Surgical Oncology, Second Affiliated Hospital, Zhejiang University School of Medicine, Hangzhou, People’s Republic of China

**Keywords:** Breast cancer, Immune checkpoint molecules, Prognostic value

## Abstract

Immune checkpoint blockade treatments bring remarkable clinical benefits to fighting several solid malignancies. However, the efficacy of immune checkpoint blockade in breast cancer remains controversial. Several clinical trials of immune checkpoint blockades focused on the effect of CTLA4 and PD1/PDL1 checkpoint inhibitors on breast cancer. Only a small portion of patients benefited from these therapies. Here we systematically investigated the expression of 50 immune checkpoint genes, including ADORA2A, LAG-3, TIM-3, PD1, PDL1, PDL2, CTLA-4, IDO1, B7-H3, B7-H4, CD244, BTLA, TIGIT, CD80, CD86, VISTA, CD28, ICOS, ICOSLG, HVEM, CD160, LIGHT, CD137, CD137L, OX40, CD70, CD27, CD40, CD40LG, LGALS9, GITRL, CEACAM1, CD47, SIRPA, DNAM1, CD155, 2B4, CD48, TMIGD2, HHLA2, BTN2A1, DC-SIGN, BTN2A2, BTN3A1, BTNL3, BTNL9, CD96, TDO, CD200 and CD200R, in different subtypes of breast cancer and assessed their prognostic value. The results showed that the expression patterns of these 50 immune checkpoint genes were distinct in breast cancer. High expression of B7-H3 mRNA was significantly associated with worse overall survival (OS), especially in patients with luminal A and luminal B breast cancer. The mRNA expression levels of TIM-3, ADORA2A, LAG3, CD86, CD80, PD1 and IDO1 had no relationship with OS in breast cancer. High expression levels of CTLA-4 and TIGIT were correlated with favorable prognosis in breast cancer. Interestingly, we observed that B7-H3 expression was negatively correlated with the efficacy of cyclophosphamide (CTX). In summary, our study suggested that B7-H3 has potential prognostic value in breast cancer and is a promising target for immune therapy.

## Introduction

Cancer immunotherapy is emerging as a very promising strategy [1]. Cancer immunotherapy includes active vaccination, adoptive cell transfer therapy and immune checkpoint blockade. Among these treatments, immune checkpoint blockade stands out as having remarkable clinical benefits for patients with melanoma, renal carcinoma, non-small-cell lung cancer and other solid tumors [2].

Immune checkpoints refer to various immune inhibitory pathways that maintain self-tolerance and modulate the duration and amplitude of immune responses in physical condition [3]. In patients with cancers, tumor cells can hijack certain immune checkpoint pathways, escape immune surveillance and resist the cytotoxic effect of host T cells [1]. Thus, immune checkpoint blockade can reduce the immune escape of tumor cells and limit tumor growth. Multiple immune checkpoint pathways have already been reported. The best characterized pathways are the interactions between CTLA-4 and CD80/86, and the binding of PD-1 to PD-L1 to tumor cells [4–6]. Both pathways can inhibit the proliferation and function of T cells, causing immune evasion. Numerous promising immune checkpoints have been reported including B7 family inhibitory ligands, such as B7-H3 (CD276), B7-H4 (VCTN1), LAG3, CD244, BTLA (CD272), TIM3 (HAVcr2), TIGIT, VISTA, IDO1 and ADORA2 [7]. Preclinical mouse models of cancer have shown that blockade of many of these individual immune-checkpoint ligands or receptors can enhance anti-tumor immunity.

Breast cancer was previously not considered an ideal model for immunotherapy because it was suspected to be immunologically silent. Recently, an increasing number of studies have found that some breast tumors are in fact sometimes heavily infiltrated by immune cells. Therefore, several clinical trials had been developed to investigate the efficacy and safety of immune checkpoint inhibitors in patients with breast cancer, such as HER2 amplified tumor and triple negative breast cancer (TNBC). However, most of these studies focused on the effect of PD-1 and CTLA4 inhibitors on breast cancer, and the overall response rate (ORR) was relatively low. Interestingly, a study performed by Jia et al. investigated the roles of three immune checkpoint molecules (IDO, PD-1, PD-L1) in head and neck cancer [8]. They found that higher expression of IDO was connected with poorer overall survival (OS) in head and neck cancer patients, whereas PD-1 had no relationship with prognosis. Therefore, these data indicated that different immune checkpoints may play different roles in different types of cancers. It raised our interest in investigating the prognostic value of all the immune checkpoint genes in breast cancer.

Here, we selected 50 genes that were reported to encode proteins which function as immune checkpoints, including ADORA2A, LAG-3, TIM-3, PD1, PDL1, PDL2, CTLA-4, IDO1, B7-H3, B7-H4, CD244, BTLA, TIGIT, CD80, CD86, VISTA, CD28, ICOS, ICOSLG, HVEM, CD160, LIGHT, CD137, CD137L, OX40, CD70, CD27, CD40, CD40LG, LGALS9, GITRL, CEACAM1, CD47, SIRPA, DNAM1, CD155, 2B4, CD48, TMIGD2, HHLA2, BTN2A1, DC-SIGN, BTN2A2, BTN3A1, BTNL3, BTNL9, CD96, TDO, CD200 and CD200R. Using UALCAN database, we compared the expression levels of mRNAs in normal tissue and different subtypes of breast cancer. The ‘Kaplan–Meier plotter’ (KM-plotter) database was used to analyze the association between gene mRNA expression and prognosis of breast cancer. Additionally, we analyzed the correlation between mRNA expression of these immune checkpoint genes with the efficiency of clinically used therapeutic agents (endocrine, chemotherapeutic and targeted agents) for breast cancer.

## Materials and methods

### Study design

We examined the mRNA expression level of candidate immune checkpoint genes between breast cancer and normal tissue based on data from the UALCAN database (http://ualcan.path.uab.edu/). A heat map was made to compare the expression differences in these two groups. The relationship between immune checkpoint mRNA expression and prognosis in breast cancer was analyzed with the KM-plotter database (http://www.kmplot.com//). Moreover, the CellMiner60 website was used to assess the association between immune checkpoint mRNA expression and the efficiency of several clinic drugs, which were widely used for breast cancer (https://discover.nci.nih.gov/cellminer/).

### UALCAN

UALCAN is a microarray database that contains TCGA raw data, including gene expression and sample information. Using this database, the mRNA expression levels of ADORA2A, LAG-3, TIM-3, PD1, PDL1, PDL2, CTLA-4, IDO1, B7-H3, B7-H4, CD244, BTLA, TIGIT, CD80, CD86 and VISTA both in breast cancer and normal breast tissue were extracted.

### KM-plotter

The KM-plotter database contains the data on gene mRNA expression and prognostic parameters. The recommended probes for candidate immune checkpoint genes were selected for analysis. The cases were divided into two groups by the median of gene mRNA expression. Clinical parameters were extracted, including estrogen receptor (ER), human epithelial growth factor receptor-2 (HER-2), p53 mutation, status of lymph nodal, grade of disease and chemotherapy history. The OS, relapse-free survival (RFS) and log-rank *P*-value were calculated.

### CellMiner analysis tool

CellMiner analysis is a tool that was designed for exploring gene transcription expression and drug sensitivity based on the NCI-60 cell line panel. The original data on drug sensitivity were downloaded as negative log10 of the concentration at which certain drugs can inhibit 50% of the cellular growth. The expression of immune checkpoints and drug sensitivity was transformed to Z-scores. Briefly, the immune checkpoint gene expression values and the drug sensitivities were obtained by subtracting the means of each and dividing them by the standard deviations. The association between immune checkpoint gene expression and drug sensitivity was estimated by linear regression analysis.

### Data analysis

The gene expression of immune checkpoints in breast cancer and normal tissue was obtained from the UALCAN database. The median with 90% intervals of expression level were extracted and normalized to the expression of normal tissue. Heat maps were used to show the differential expression of several immune checkpoints. In the analysis of the prognostic value of immune checkpoint genes, the cases were divided into two groups by the median expression level. The significant difference in the prognosis between the two groups was analyzed using the Kaplan–Meier method. Cox regression analysis was used to estimate the hazard ratios (HRs), and log-rank *P*-values were calculated. The *P*-value was calculated with SPSS version 24. *P*-values <0.05 were considered statistically significant.

## Results

### The mRNA expression pattern of immune checkpoint genes in breast cancer and normal tissue

**Figure 1:**
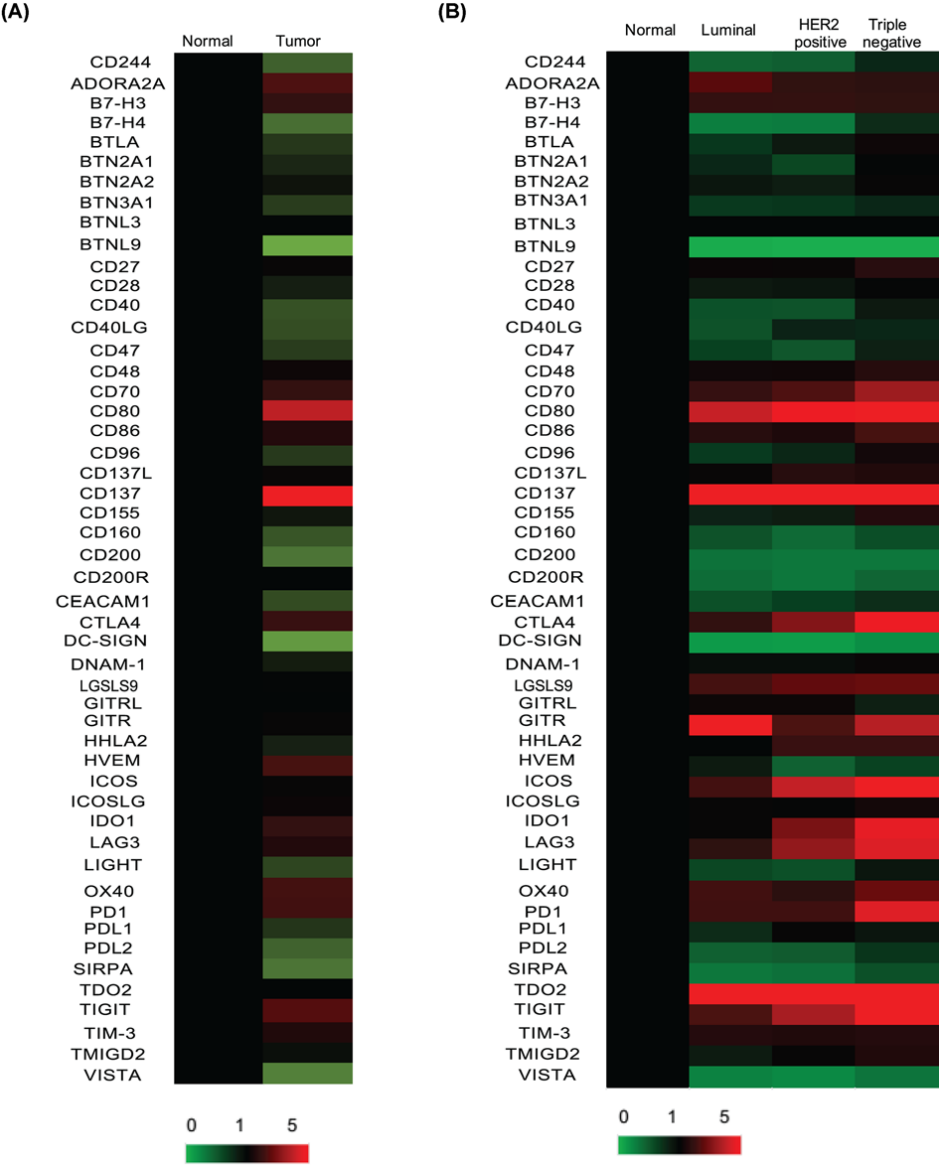
The heat map of immune checkpoint genes in breast cancer and normal tissue (**A**) The mRNA expression of 50 immune checkpoint genes in breast cancer and normal tissue. (**B**) The mRNA expression of 50 immune checkpoint genes in different subtype of breast cancer and normal tissue.

Fifty immune checkpoint genes were assessed by the UALCAN database, including ADORA2A, LAG-3, TIM-3, PD1, PDL1, PDL2, CTLA-4, IDO1, B7-H3, B7-H4, CD244, BTLA, TIGIT, CD80, CD86, VISTA, CD28, ICOS, ICOSLG, HVEM, CD160, LIGHT, CD137, CD137L, OX40, CD70, CD27, CD40, CD40LG, LGALS9, GITRL, CEACAM1, CD47, SIRPA, DNAM1, CD155, 2B4, CD48, TMIGD2, HHLA2, BTN2A1, DC-SIGN, BTN2A2, BTN3A1, BTNL3, BTNL9, CD96, TDO, CD200 and CD200R. The mean mRNA expression of these genes both in breast cancer and normal breast tissue was obtained and analyzed by heat map (Figure 1A). The results showed that the mRNA expression patterns of immune checkpoint genes in breast cancer were quite different. Compared with normal tissue, CD244, B7-H4, BTLA, BTN2A1, BTN3A1, BTNL9, CD28, CD40, CD40LG, CD47, CD96, CD160, CD200, CEACAM1, DC-SIGN, LIGHT, SIRPA, PDL1, PDL2 and VISTA were down-regulated in breast cancer, whereas ADORA2A, B7-H3, CD70, CD80, CD86, CD137, CTLA-4, HVEM, IDO1, LAG-3, OX40, PD1 and TIGIT were up-regulated in breast cancer. We further performed subgroup analysis based on different subtypes of breast cancer. The results showed that CTLA-4, IDO1, LAG-3, TIGIT and PD1 were specifically increased most in TBNC among different types of cancer. ADORA2A was observed to be highly expressed in luminal breast cancer (Figure 1B). These data indicated that the expression patterns of immune checkpoint genes were distinct in breast cancer.

### The prognostic value of immune checkpoint genes in breast cancer

**Figure 2:**
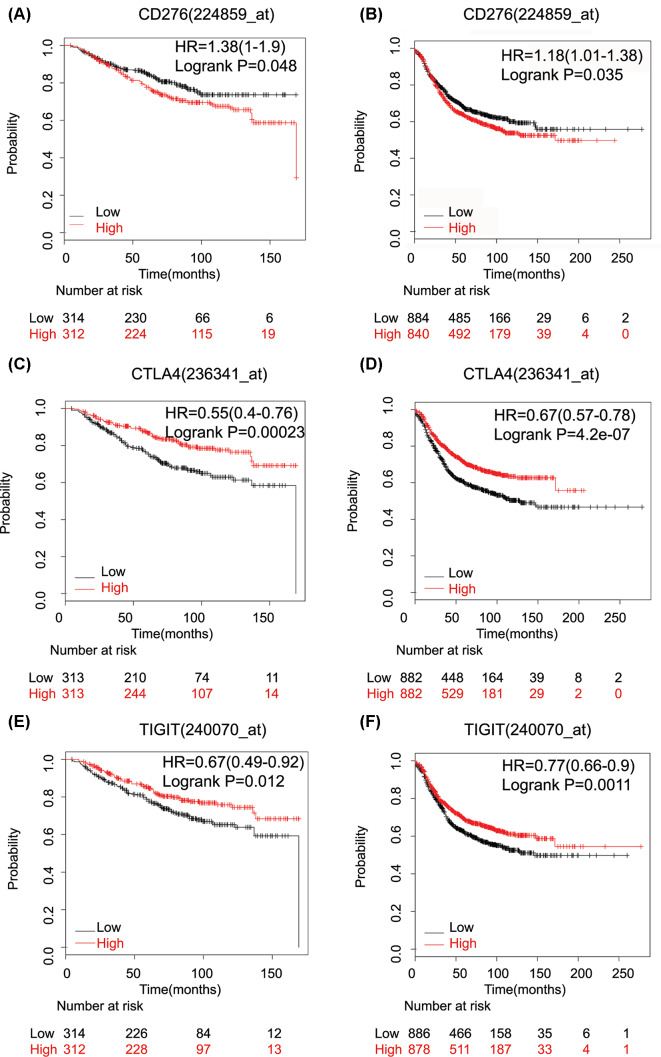
The correlation between inhibitory immune checkpoint genes mRNA expression and prognosis of breast cancer patients (**A,B**) The B7-H3 (CD276) mRNA expression is associated with a worse OS (A) and RFS (B) in breast cancer patients. (**C,D**) The CTLA4 mRNA expression is associated with a better OS (C) and RFS (D) in breast cancer patients. (**E,F**) The TIGIT mRNA expression is associated with a better OS (E) and RFS (F) in breast cancer patients.

Next, we investigated the prognostic value of ten up-regulated inhibitory immune checkpoint genes, ADORA2A, LAG-3, TIM-3, PD1, CTLA-4, IDO1, B7-H3, TIGIT, CD80 and CD86, and six down-regulated costimulatory checkpoint genes, CD28, CD40, CD40LG, CD160, CD200 and LIGHT (Table 1). The results showed that high mRNA expression of costimulatory checkpoint genes was associated with longer RFS (CD28: HR = 0.71 (0.64–0.79), *P*=1.1E-09; CD160: HR = 0.75 (0.67–0.84), *P*=2.80E-07; LIGHT: HR = 0.84 (0.76–0.94), *P*=0.0024; CD40: HR = 0.81 (0.73–0.9), *P*=0.00017; CD40LG: HR = 0.8 (0.71–0.89), *P*=3.90E-05; CD200: HR = 0.82 (0.73–0.91), *P*=0.00034). On the other hand, high expression of B7-H3 mRNA was significantly associated with worse OS (HR: 1.38 (1.00–1.90); *P*=0.048) (Figure 2A, Table 1) and RFS (HR: 1.18 (1.01–1.38); *P*=0.035) (Figure 2B) in breast cancer cases. The mRNA expression of TIM-3, ADORA2A, LAG3, CD86, CD80, PD1 and IDO1 had no relationship with OS in breast cancer (Table 1). ADORA2A, LAG3, PD1 and IDO1 mRNA expression had significant favorable associations with RFS (pooled HRs: HR: 0.83 (0.75-0.93); *P*=0.00093), (HR: 0.88 (0.79-0.99); *P*=0.026), (HR: 0.74 (0.67-0.83); *P*=8.5E-08) and (HR: 0.89 (0.8–0.99); *P*=0.034), respectively). CTLA-4 and TIGIT were correlated with better prognosis in breast cancer. High mRNA expression of CTLA-4 was associated with longer OS (HR: 0.55 (0.40–0.76); *P*=2.30E-04) (Figure 2C) and RFS (HR: 0.67 (0.57–0.78); *P*=4.20E-07) (Figure 2D) in all patients. High mRNA expression of TIGIT was significantly connected to favorable OS (HR: 0.67 (0.49–0.92); *P*=0.012) (Figure 2E) and RFS (HR: 0.77 (0.66–0.9); *P*=0.0011) (Figure 2F) in breast cancer patients. These results were also verified by GEO database (Supplementary Figure S1).

**Table 1:** The prognostic value of immune checkpoint genes in breast cancer

Gene	Affymetrix IDs	OS			RFS		
		HR	95% CI	*P*-value	HR	95% CI	*P*-value
*ADORA2A*	205013_s_at	0.91	0.74–1.13	0.4	0.83	0.75–0.93	0.00093
*LAG-3*	206486_at	0.94	0.76–1.17	0.59	0.88	0.79–0.99	0.026
*TIM-3*	235458_at	1.07	0.78–1.46	0.67	1.09	0.93–1.27	0.3
*PD1*	207634_at	0.84	0.68–1.04	0.11	0.74	0.67–0.83	8.5E-08
*CTLA-4*	236341_at	0.55	0.4–0.76	0.00023	0.67	0.57–0.78	4.2E-07
*IDO1*	210029_at	0.84	0.68–1.04	0.11	0.89	0.8–0.99	0.034
*B7-H3*	224859_at	1.38	1–1.9	0.048	1.18	1.01–1.38	0.035
*TIGIT*	240070_at	0.67	0.49–0.92	0.012	0.77	0.66–0.9	0.0011
*CD80*	1554519_at	0.84	0.61–1.14	0.26	0.9	0.77–1.05	0.19
*CD86*	205685_at	0.97	0.78–1.2	0.78	0.93	0.83–1.04	0.19
*CD28*	206545_at	0.82	0.66–1.02	0.08	0.71	0.64–0.79	1.1E-09
*CD160*	207840_at	0.88	0.71–1.09	0.26	0.75	0.67–0.84	2.80E-07
*LIGHT*	207907_at	0.94	0.76–1.17	0.6	0.84	0.76–0.94	0.0024
*CD40*	215346_at	0.81	0.65–1	0.05	0.81	0.73–0.9	0.00017
*CD40LG*	207892_at	0.83	0.67–1.03	0.096	0.8	0.71–0.89	3.90E-05
*CD200*	209583_s_at	0.85	0.69–1.06	0.15	0.82	0.73–0.91	0.00034

### The prognostic value of inhibitory immune checkpoint genes in subtypes of breast cancer

**Figure 3:**
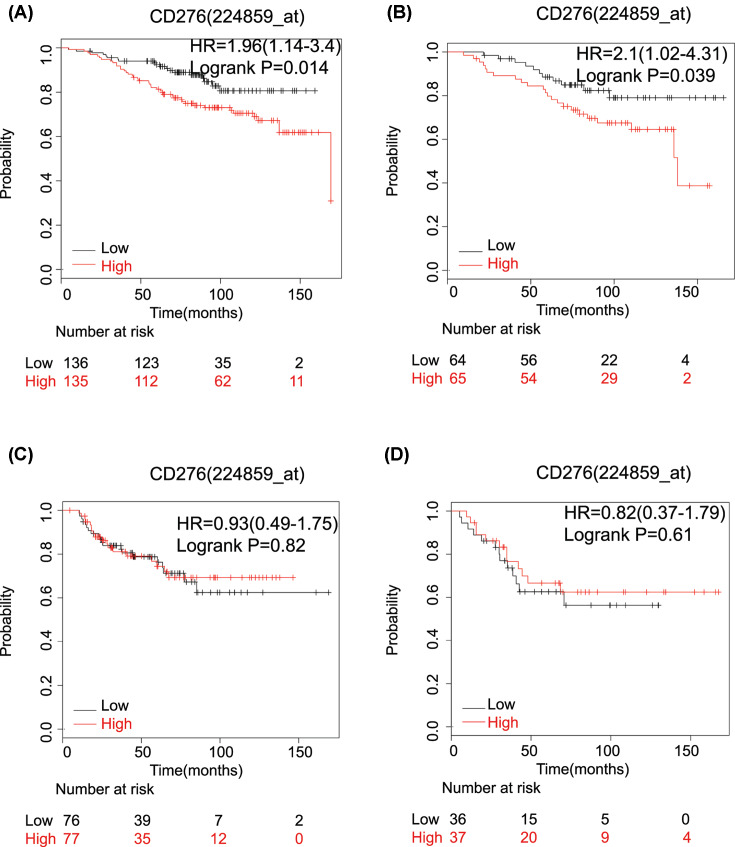
The correlation between B7-H3 mRNA expression and prognosis of different subtypes of breast cancer patients (**A**) The B7-H3(CD276) mRNA expression is associated with a worse OS in luminal A breast cancer patients; (**B**) the B7-H3 (CD276) mRNA expression is associated with a worse OS in luminal B breast cancer patients. (**C,D**) The B7-H3 (CD276) mRNA expression has no relationship with prognosis in basal breast cancer patients (C) and HER-2 amplification cases (D).

We further explored the prognostic value of inhibitory immune checkpoint genes in subtypes of breast cancer patients due to the availability of inhibitory immune checkpoint inhibitors in the clinic (Table 2). The results showed that high mRNA expression of CTLA-4 (OS: HR: 0.24 (0.12–0.50); *P*=3.80E-05), IDO1 (OS: HR: 0.44 (0.26–0.74); *P*=0.0014), LAG-3 (OS: HR: 0.37 (0.22–0.63); *P*=1.20E-04), CD86 (OS: HR: 0.5 (0.3–0.83); *P*=0.0064), CD80 (OS: HR: 0.28 (0.13–0.58); *P*=3.0E-04), TIGIT (OS: HR: 0.39 (0.2–0.77); *P*=0.0051) and TIM-3 (OS: HR: 0.52 (0.27–1.0); *P*=0.0045) was significantly associated with longer OS in TNBC. High mRNA expression of B7-H3 was associated with worse OS in patients with luminal A and luminal B breast cancer. The pooled HR was 1.96 (1.24–3.4); *P*=0.014) (Figure 3A) and 2.1 (1.02–4.31); *P*=0.039) (Figure 3B), respectively. No prognostic value was found in basal-like and HER-2 amplification breast cancer cases (Figure 3C,D). The elevated average expression of TIGIT was significantly associated with better OS (HR: 0.35 (0.17–0.74); *P*=0.0037) in luminal B breast cancer (Table 2). The mRNA expression of PD1 and ADORA2A had no relationship with OS in different types of breast cancer (Table 2).

**Table 2:** The prognostic value of inhibitory immune checkpoint genes in subtypes of breast cancer

Gene	Affymetrix IDs	Subtype	HR	95% CI	*P*-value
*ADORA2A*	205013_s_at	basal	0.65	0.39–1.07	0.085
		luminal A	0.96	0.68–1.36	0.82
		luminal B	0.98	0.68–1.42	0.93
		HER-2	0.87	0.46–1.66	0.67
*LAG-3*	206486_at	basal	0.37	0.22–0.63	0.00012
		luminal A	1.2	0.79–1.59	0.53
		luminal B	1.03	0.71–1.49	0.88
		HER-2	1.04	0.55–1.99	0.9
*TIM-3*	235458_at	basal	0.52	0.27–1	0.045
		luminal A	1.5	0.9–2.49	0.12
		luminal B	1.13	0.57–2.21	0.73
		HER-2	1.1	0.5–2.42	0.81
*PD1*	207634_at	basal	0.64	0.39–1.05	0.073
		luminal A	0.71	0.5–1.02	0.059
		luminal B	0.91	0.63–1.33	0.64
		HER-2	0.89	0.46–1.69	0.72
*CTLA-4*	236341_at	basal	0.24	0.12–0.5	3.8E-05
		luminal A	0.77	0.47–1.28	0.31
		luminal B	0.46	0.23–0.92	0.025
		HER-2	0.75	0.34–1.65	0.47
*IDO1*	210029_at	basal	0.44	0.26–0.74	0.0014
		luminal A	1	0.7–1.42	1
		luminal B	0.74	0.51–1.08	0.12
		HER-2	0.59	0.31–1.16	0.12
*B7-H3*	224859_at	basal	0.93	0.49–1.75	0.82
		luminal A	1.96	1.24–3.4	0.014
		luminal B	2.1	1.02–4.31	0.039
		HER-2	0.82	0.37–1.79	0.61
*TIGIT*	240070_at	basal	0.39	0.2–0.77	0.0051
		luminal A	0.82	0.5–1.37	0.45
		luminal B	0.35	0.17–0.74	0.0037
		HER-2	0.54	0.24–1.2	0.12
*CD80*	1554519_at	basal	0.28	0.13–0.58	0.0003
		luminal A	1.5	0.9–2.49	0.11
		luminal B	1.11	0.57–2.18	0.76
		HER-2	0.64	0.29–1.42	0.27
*CD86*	205685_at	basal	0.5	0.3–0.83	0.0064
		luminal A	0.91	0.64–1.29	0.6
		luminal B	0.76	0.52–1.1	0.14
		HER-2	0.72	0.38–1.38	0.32

### Relationship between inhibitory immune checkpoint genes and clinicopathological characteristics

The relationship between the clinicopathological characteristics and inhibitory immune checkpoint gene expression levels was also investigated. The results showed that a better OS was found in grade III patients with high expression of CTLA-4 (HR: 0.43 (0.25–0.73); *P*=0.0013), LAG-3 (HR: 0.68 (0.48–0.94); *P*=0.019) and IDO1 (HR: 0.53 (0.37–0.74); *P*=2.10E-04) than that in grade I and II patients. In addition, we observed that the prognostic value of IDO1 and TIM-3 was distinct in the patients with different ER statuses. High mRNA expression of IDO1 was correlated with longer OS in ER-negative patients (HR: 0.63 (0.39–1.0); *P*=0.046) but not in ER-positive patients (HR: 1.04 (0.73–1.48); *P*=0.82). Similarly, TIM-3 only showed better OS in ER-negative patients (HR: 0.45 (0.21–0.93); *P*=0.029). Additionally, IDO1 expression was correlated with better OS in HER-2 amplified patients (HR: 0.46 (0.22–0.95); *P*=0.032) but not in HER-2 negative patients (HR: 1.01 (0.43–2.39); *P*=0.98). CTLA-4 expression was associated with longer OS in lymph node-positive patients (OS: HR: 0.53 (0.31–0.92); *P*=0.021) but not in lymph node-negative patients (HR: 0.51 (0.20–1.29); *P*=0.14). The prognostic values of IDO1, LAG-3, ADORA2A, B7-H3 and PD1 were not affected by chemotherapy history and p53 mutation (Table 3).

**Table 3:** Relationship between inhibitory immune checkpoint genes and clinicopathological characteristics

Gene	Affymetrix IDs	Clinicopathological characteristics		HR	95% CI	*P*-value
*ADORA2A*	205013_s_at	Grade	I	0.86	0.34–2.18	0.75
			II	1.17	0.76–1.79	0.47
			III	1	0.72–1.38	0.99
		ER	positive	1.1	0.77–1.57	0.6
			negative	0.98	0.62–1.54	0.94
		HER-2	positive	0.77	0.38–1.55	0.46
			negative	0.46	0.18–1.2	0.1
		Chemotherapy history	yes	0.76	0.46–1.23	0.26
			no	1	0.71–1.42	0.98
		p53 status	mutant	1.2	0.56–2.57	0.64
			wild-type	1.67	0.86–3.25	0.13
*LAG-3*	206486_at	Grade	I	0.97	0.39–2.4	0.94
			II	1.27	0.83–1.94	0.28
			III	0.68	0.48–0.94	0.019
		ER	positive	1.18	0.83–1.69	0.35
			negative	0.7	0.44–1.11	0.12
		HER-2	positive	0.79	0.39–1.59	0.51
			negative	0.66	0.28–1.57	0.34
		Chemotherapy history	yes	0.71	0.44–1.17	0.18
			no	1.18	0.83–1.67	0.35
		p53 status	mutant	0.73	0.34–1.56	0.41
			wild-type	1.52	0.79–2.93	0.21
*TIM-3*	235458_at	Grade	I	0.65	0.17–2.48	0.64
			II	1.25	0.4–3.96	0.7
			III	0.66	0.4–1.1	0.11
		ER	positive	1.63	0.76–3.52	0.21
			negative	0.45	0.21–0.94	0.029
		HER-2	positive	/	/	/
			negative	0.92	0.32–2.62	0.87
		Chemotherapy history	yes	0.78	0.28–2.16	0.63
			no	/	/	/
		p53 status	mutant	0.65	0.17–2.48	0.53
			wild-type	/	/	/
*PD1*	207634_at	Grade	I	0.54	0.21–1.36	0.18
			II	1.07	0.7–1.65	0.75
			III	0.84	0.61–1.17	0.3
		ER	positive	0.93	0.65–1.33	0.69
			negative	0.71	0.45–1.12	0.14
		HER-2	positive	1.03	0.5–2.1	0.94
			negative	0.46	0.16–1.27	0.12
		Chemotherapy history	yes	1.35	0.81–2.25	0.24
			no	0.77	0.53–1.1	0.14
		p53 status	mutant	1.47	0.63–3.43)	0.37
			wild-type	1.08	0.57–2.06)	0.81
*CTLA-4*	236341_at	Grade	I	0.44	0.04–4.86	0.49
			II	1.11	0.36–3.44	0.86
			III	0.43	0.25–0.73	0.0013
		ER	positive	0.86	0.41–1.84	0.7
			negative	0.56	0.27–1.16	0.12
		HER-2	positive	/	/	/
			negative	0.73	0.25–2.09	0.55
		Chemotherapy history	yes	0.31	0.1–0.96)	0.032
			no	/	/	/
		p53 status	mutant	0.25	0.05–1.18	0.058
			wild-type	/	/	/
IDO1	210029_at	Grade	I	0.56	0.2–1.41	0.21
			II	1.39	0.9–2.13	0.134
			III	0.53	0.37–0.74	0.00021
		ER	positive	1.04	0.73–1.48	0.82
			negative	0.63	0.39–1	0.046
		HER-2	positive	0.46	0.22–0.95	0.032
			negative	1.01	0.43–2.39	0.98
		Chemotherapy history	yes	0.78	0.48–1.28	0.33
			no	1.07	0.75–1.51	0.72
		p53 status	mutant	0.81	0.38–1.74	0.59
			wild-type	1.15	0.6–2.19	0.67
B7-H3	224859_at	Grade	I	2.14	0.19–23.79	0.53
			II	1.16	0.38–3.61	0.79
			III	1.34	0.8–2.23	0.25
		ER	positive	2.13	0.96–4.73	0.059
			negative	1.11	0.55–2.25	0.77
		HER-2	positive	/	/	/
			negative	2.16	0.72–6.48	0.16
		Chemotherapy history	yes	1.04	0.38–2.87	0.94
			no	/	/	/
		p53 status	mutant	1.15	0.31–4.29	0.84
			wild-type	/	/	/
TIGIT	240070_at	Grade	I	/	/	/
			II	2.48	0.75–8.26	0.13
			III	0.55	0.32–0.92	0.022
		ER	positive	1.07	0.5–2.28	0.85
			negative	0.84	0.42-1.71	0.64
		HER-2	positive	/	/	/
			negative	0.71	0.25–2.06	0.53
		Chemotherapy history	yes	0.45	0.15–1.32	0.13
			no	/	/	/
		p53 status	mutant	0.48	0.12–1.92	0.29
			wild-type	/	/	/
CD80	1554519_at	Grade	I	/	/	/
			II	0.77	0.24–2.42	0.65
			III	0.34	0.19–0.61	0.00013
		ER	positive	0.76	0.36–1.63	0.48
			negative	0.38	0.18–0.8	0.0087
		HER-2	positive	/	/	/
			negative	0.53	0.18–1.59	0.25
		Chemotherapy history	yes	0.52	0.81–1.51	0.22
			no	/	/	/
		p53 status	mutant	0.46	0.11–1.84	0.26
			wild-type	/	/	/
CD86	205685_at	Grade	I	0.91	0.37–2.24	0.84
			II	0.92	0.6–1.42	0.72
			III	0.79	0.57–1.1	0.16
		ER	positive	0.9	0.63–1.28	0.57
			negative	1.09	0.7–1.72	0.7
		HER-2	positive	0.79	0.39–1.58	0.5
			negative	0.97	0.41–2.28	0.94
		Chemotherapy history	yes	0.91	0.56–1.48	0.69
			no	1.01	0.72–1.44	0.93
		p53 status	mutant	0.61	0.28–1.32	0.21
			wild-type	1.12	0.59–2.14	0.73

### Correlations between inhibitory immune checkpoint gene expression and drug sensitivity

**Figure 4:**
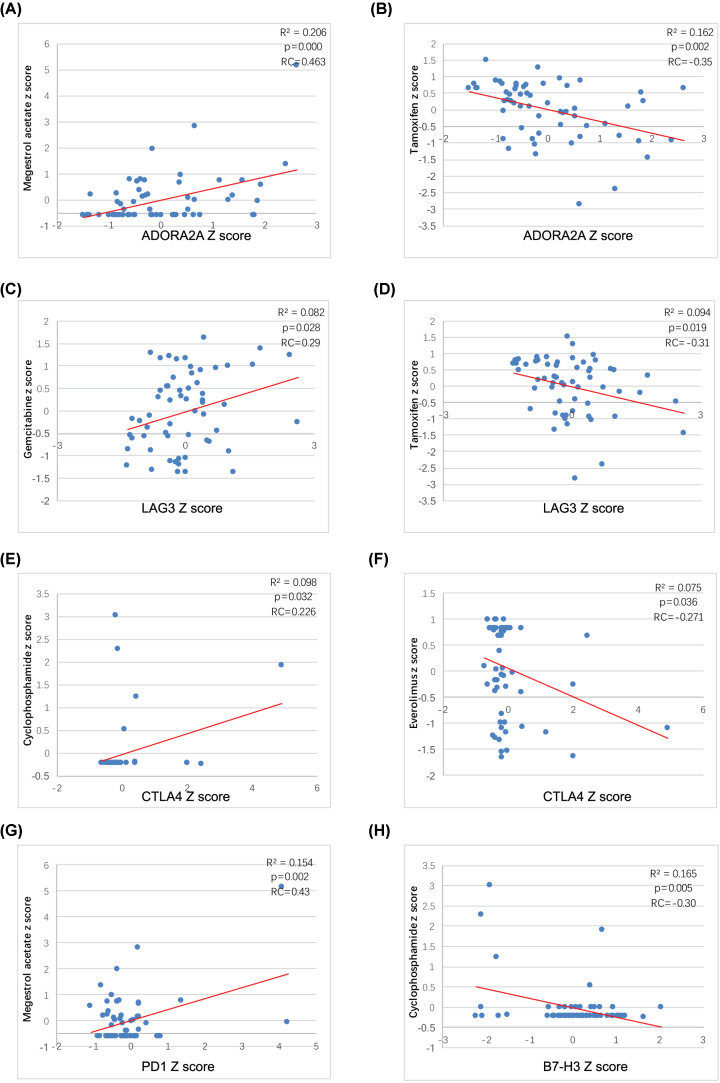
Association between inhibitory immune checkpoint genes mRNA and sensitivity of different cell lines to target drugs The regression figures of ADORA2A mRNA and efficacy of megestrol acetate (**A**), ADORA2A mRNA and efficacy of Tamoxifen (**B**), LAG-3 expression and efficacy of gemcitabine (**C**), LAG-3 expression and efficacy of Tamoxifen (**D**), CTLA-4 expression and efficacy of CTX (**E**), CTLA-4 expression and efficacy of everolimus (**F**), PD1 expression and efficacy of megestrol acetate (**G**), BT-H3 expression and efficacy of CTX (**H**). The data were transformed to Z-scores and were downloaded from Cell Miner Tools.

The correlation between ADORA2A, LAG-3, CTLA-4, PD1 and B7-H3 expression and drug efficiency was investigated. First, we observed that ADORA2A mRNA expression was associated with the sensitivity of two endocrine drugs, tamoxifen and megestrol acetate. Tumors with higher expression of ADORA2A mRNA were more sensitive to megestrol acetate (Figure 4A) but less sensitive to Tamoxifen (Figure 4B). Second, LAG-3 expression was found to be correlated with gemcitabine sensitivity (Figure 4C) and Tamoxifen resistance (Figure 4D). Third, CTLA-4 expression was connected to cyclophosphamide (CTX) sensitivity (Figure 4E) and everolimus resistance (Figure 4F). In addition, the results showed that PD1 expression was associated with the susceptibility of tumor cells to megestrol acetate (Figure 4G). Last, BT-H3 expression was correlated with the efficacy of CTX (Figure 4H).

## Discussion

Immune checkpoint blockade is emerging as the most promising strategy for tumor treatment. Large benefits from checkpoint blockade have been demonstrated in several cancer subtypes, including melanoma, lung, bladder, kidney, head and neck carcinoma and Hodgkin’s disease [2,9–12]. However, these benefits remain controversial in breast cancer. Several clinical trials have investigated the responses of immune checkpoint inhibitors in metastatic breast cancer [13]. The KEYNOTE 028 trial has investigated the ORR of the PD1 inhibitor pembrolizumab in HR+ HER2− metastatic breast cancer patients. The results demonstrated that the ORR was just 12% [14]. The PANACEA (KEYNOTE-014) Phase Ib/II trial investigated the efficacy of pembrolizumab combined with trastuzumab in trastuzumab-resistant metastatic breast cancer, and the ORR was 15%. In another trial, the researchers investigated the ORR of pembrolizumab in patients with PDL1-positive, chemotherapy-resistant metastatic TNBC [15], with an ORR of 18.5%. From these data, we found that most studies focused on the effect of CTLA4 and PD1/PDL1 checkpoint inhibitors on breast cancer, and only a small portion of patients responded to these treatments [16]. Therefore, it is urgent to explore additional co-inhibitory molecules for breast cancer immunotherapy. Few promising immune checkpoints have been investigated, including B7-H3, B7-H4, LAG3, CD244, BTLA, TIM3, TIGIT, VISTA, IDO1 and ADORA2.

In the present study, we systematically analyzed the prognostic value of immune checkpoint molecules. We first assessed the expression levels of 50 immune checkpoints genes, including ADORA2A, LAG-3, TIM-3, PD1, PDL1, PDL2, CTLA-4, IDO1, B7-H3, B7-H4, CD244, BTLA, TIGIT, CD80, CD86, VISTA, CD28, ICOS, ICOSLG, HVEM, CD160, LIGHT, CD137, CD137L, OX40, CD70, CD27, CD40, CD40LG, LGALS9, GITRL, CEACAM1, CD47, SIRPA, DNAM1, CD155, 2B4, CD48, TMIGD2, HHLA2, BTN2A1, DC-SIGN, BTN2A2, BTN3A1, BTNL3, BTNL9, CD96, TDO, CD200 and CD200R. The results showed that the expression patterns of immune checkpoint genes were distinct in breast cancer. Second, we analyzed the prognostic value of ten up-regulated inhibitory immune checkpoint genes, including ADORA2A, LAG-3, TIM-3, PD1, CTLA-4, IDO1, B7-H3, TIGIT, CD80 and CD86. We found that only high expression of B7-H3 mRNA was significantly associated with worse OS and RFS in breast cancer cases. CTLA-4 and TIGIT were related to longer OS and RFS in breast cancer patients. ADORA2A, LAG3, PD1 and IDO1 mRNA expression was connected to favorable associations with RFS. These results are in line with a previous study that also reported that PD1, LAG3 and CTLA4 expression predicted significant, favorable prognosis [17]. Third, we performed subgroup analysis based on the subtype of breast cancer. The results showed that high mRNA expression of CTLA-4, IDO1, LAG-3, CD86, CD80, TIGIT and TIM-3 was significantly associated with longer OS in TNBC. The high mRNA expression of B7-H3 was associated with worse OS in patients with luminal A and luminal B breast cancer. B7-H3 belongs to the B7 family cosignaling molecules that are mainly expressed by APCs and tumor cells. B7-H3 was reported to be overexpressed in various human cancers [18]. Five-year survival analysis of breast cancer patients suggested that high expression of B7-H3 was correlated with poor prognosis [19]. In addition, high B7-H3 expression was reported to be related to unfavorable clinicopathological parameters in breast cancer [20]. The mechanisms of B7-H3 involved in cancer progression include binding to MVP, which regulates the activation of the MAPK kinase pathway and subsequently regulates breast cancer stem cell enrichment [18]. Another study also reported that high B7-H3 expression in circulating epithelial tumor cells in breast cancer patients promotes the metastasis of breast cancer [21]. Hence, B7-H3 seems to be a promising target for anticancer therapies. Lee et al. proved that inhibition of the B7-H3 immune checkpoint can effectively limit tumor growth [16]. Fourth, we investigated the relationship between the expression of inhibitory immune checkpoints and clinicopathological characteristics. The results showed better OS in grade III patients with high expression of CTLA-4, LAG-3 and IDO1 than in grade I and II patients. The high mRNA expression of IDO1 and TIM-3 was only correlated with longer OS in ER-negative patients. Additionally, IDO1 expression was correlated with better OS in HER-2-amplified patients. Last, we assessed the association between mRNA expression levels of these immune checkpoints and efficacies of several clinic drugs. Several studies reported that checkpoint blockade strategies combined with chemotherapy appear to be more effective in breast cancer. It has been reported that Pembroluzumab combined with Eribuline in metastatic TNBC reported a 33.3% ORR in the patients evaluated. We observed a negative correlation between B7-H3 mRNA expression and sensitivity of CTX. Similar findings were reported that B7-H3 promotes Oxaliplatin resistance via upregulating the expression of X-ray repair cross complementing group 1 in cancer cells [22]. Additionally, we found a positive correlation between PD1 mRNA expression and efficacy of Megestrol acetate. Tumor cells with high CTLA-4 mRNA are more sensitive to CTX but less sensitive to Everolimus. Previous studies indicated that metronomic CTX can be immune-stimulating [23,24]. Metronomic CTX before CTLA-4 inhibitor treatment may be a reasonable choice. Everolimus is an inhibitor of mammalian target of rapamycin (mTOR), but it also works as an immunosuppressant for preventing organ transplant rejection. Everolimus may further enhance the level of immune suppression by increasing the ratio of Tregs in tumors. Hence, the tumor cells with high CTLA-4 mRNA expression may be resistant to Everolimus. We also observed that tumors with higher LAG-3 mRNA expression are less sensitive to Tamoxifen but more sensitive to gemcitabine.

Taken together, the present study indicated that the expression patterns of immune checkpoint genes were distinct and different immune checkpoint molecules correlated with different prognoses in breast cancer patients. High expression of B7-H3 mRNA was significantly associated with worse OS, especially in the patients with luminal A and luminal B breast cancer. The mRNA expression of TIM-3, ADORA2A, LAG3, CD86, CD80, PD1 and IDO1 had no relationship with OS in breast cancer. CTLA-4 and TIGIT were correlated with better prognosis in breast cancer. Interestingly, we observed that B7-H3 expression was negatively correlated with CTX efficacy. In summary, our study suggested that the immune checkpoint molecule B7-H3 has potential prognostic value and applicability to immune therapy for breast cancer. However, there is still a lack of clinical trials with high-level evidence. It is expected that more investigators will propose well-designed, prospective clinical studies to investigate the role of B7-H3 in breast cancer.

## Supplementary Material

Supplementary Figure S1

## Abbreviations

APC: antigen-presenting Cells
CTX: cyclophosphamide
ER: estrogen receptor
GEO: gene expression omnibus
HER-2: human epithelial growth factor receptor-2
HR: hazard ratio
KM-plotter: Kaplan–Meier plotter
MVP: major vault protein
ORR: overall response rate
OS: overall survival
RFS: relapse-free survival
TNBC: triple negative breast cancer
TCGA: The Cancer Genome Atlas
