# Supplementary material for: Prognostic value of immune checkpoint molecules in breast cancer
Source: Biosci Rep. 2020 Jul 7;40(7):BSR20201054. doi: 10.1042/BSR20201054 (PMC7340863; doi:10.1042/BSR20201054)
Supplement: Supplementary Figure S1 [file BSR-2020-1054_supp.pdf]

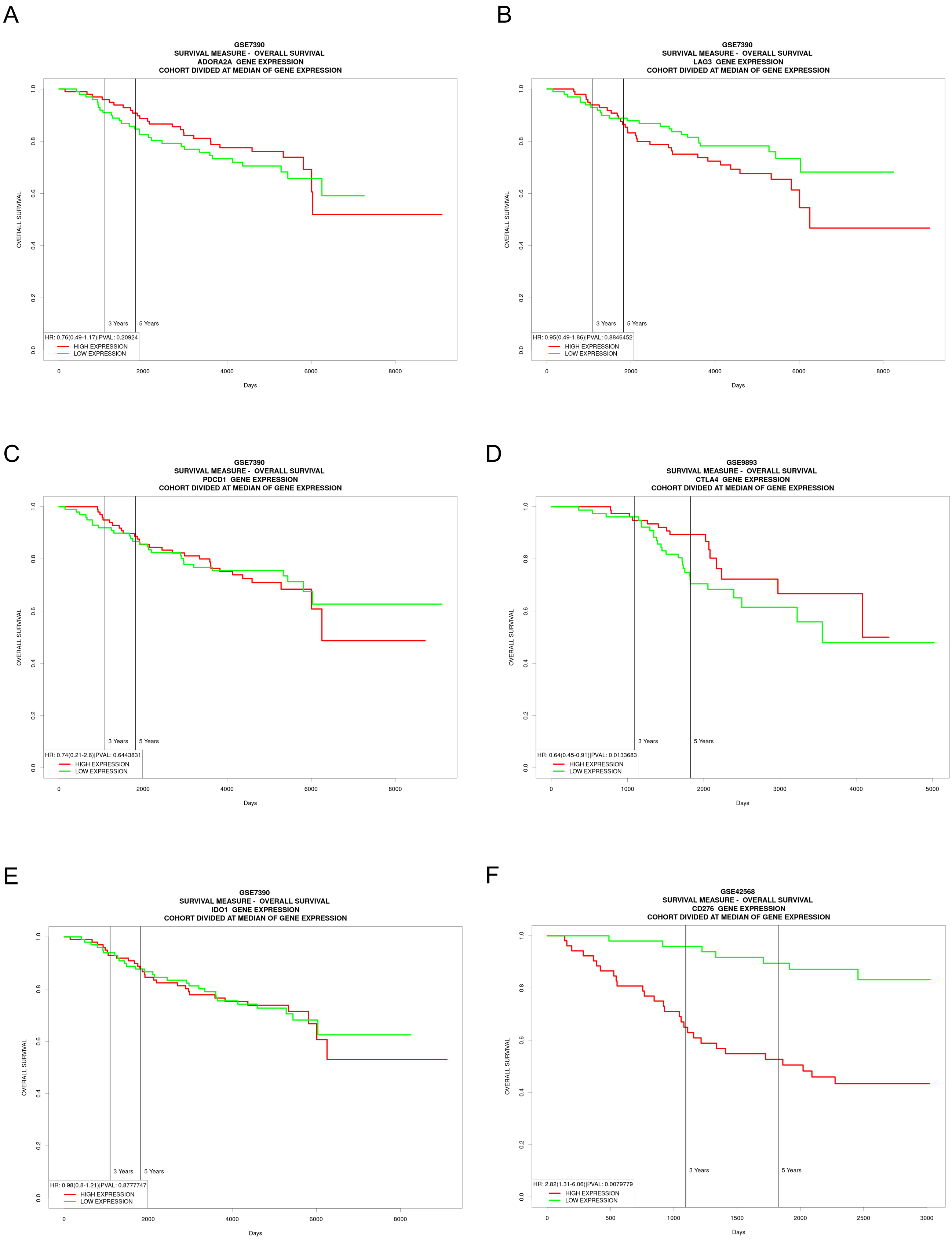
A
GSE7390
SURVIVAL MEASURE - OVERALL SURVIVAL
ADORA2A GENE EXPRESSION
COHORT DIVIDED AT MEDIAN OF GENE EXPRESSION
OVERALL SURVIVAL
0.0
0.2
0.4
0.6
0.8
1.0
3 Years
5 Years
HR: 0.76(0.49-1.17)|PVAL: 0.20924
HIGH EXPRESSION
LOW EXPRESSION
0
2000
4000
6000
8000
Days
B
GSE7390
SURVIVAL MEASURE - OVERALL SURVIVAL
LAG3 GENE EXPRESSION
COHORT DIVIDED AT MEDIAN OF GENE EXPRESSION
OVERALL SURVIVAL
0.0
0.2
0.4
0.6
0.8
1.0
3 Years
5 Years
HR: 0.95(0.49-1.86)|PVAL: 0.8846452
HIGH EXPRESSION
LOW EXPRESSION
0
2000
4000
6000
8000
Days
C
GSE7390
SURVIVAL MEASURE - OVERALL SURVIVAL
PDCD1 GENE EXPRESSION
COHORT DIVIDED AT MEDIAN OF GENE EXPRESSION
OVERALL SURVIVAL
0.0
0.2
0.4
0.6
0.8
1.0
3 Years
5 Years
HR: 0.74(0.21-2.6)|PVAL: 0.6443831
HIGH EXPRESSION
LOW EXPRESSION
0
2000
4000
6000
8000
Days
D
GSE9893
SURVIVAL MEASURE - OVERALL SURVIVAL
CTLA4 GENE EXPRESSION
COHORT DIVIDED AT MEDIAN OF GENE EXPRESSION
OVERALL SURVIVAL
0.0
0.2
0.4
0.6
0.8
1.0
3 Years
5 Years
HR: 0.64(0.45-0.91)|PVAL: 0.0133683
HIGH EXPRESSION
LOW EXPRESSION
0
1000
2000
3000
4000
5000
Days
E
GSE7390
SURVIVAL MEASURE - OVERALL SURVIVAL
IDO1 GENE EXPRESSION
COHORT DIVIDED AT MEDIAN OF GENE EXPRESSION
OVERALL SURVIVAL
0.0
0.2
0.4
0.6
0.8
1.0
3 Years
5 Years
HR: 0.98(0.8-1.21)|PVAL: 0.8777747
HIGH EXPRESSION
LOW EXPRESSION
0
2000
4000
6000
8000
Days
F
GSE42568
SURVIVAL MEASURE - OVERALL SURVIVAL
CD276 GENE EXPRESSION
COHORT DIVIDED AT MEDIAN OF GENE EXPRESSION
OVERALL SURVIVAL
0.0
0.2
0.4
0.6
0.8
1.0
3 Years
5 Years
HR: 2.82(1.31-6.06)|PVAL: 0.0079779
HIGH EXPRESSION
LOW EXPRESSION
0
500
1000
1500
2000
2500
3000
Days

Supplementary Figure S1 The correlation between immune checkpoint genes and prognosis of breast cancer patients verified by GEO database. (A) The ADORA2A mRNA expression has no relationship with OS (B) The LAG3 mRNA expression has no relationship with OS; (C) The PDCD1 mRNA expression has no relationship with OS; (D) The CTLA4 mRNA expression is associated with a better OS; (E)The IDO1 mRNA expression has no relationship with OS; (F) The B7-H3(CD276) mRNA expression is associated with a worse OS.

.
